# TILRR Promotes Migration of Immune Cells Through Induction of Soluble Inflammatory Mediators

**DOI:** 10.3389/fcell.2020.00563

**Published:** 2020-07-03

**Authors:** Mohammad Abul Kashem, Xiaoou Ren, Hongzhao Li, Binhua Liang, Lin Li, Francis Lin, Francis A. Plummer, Ma Luo

**Affiliations:** ^1^Department of Medical Microbiology and Infectious Diseases, University of Manitoba, Winnipeg, MB, Canada; ^2^JC Wilt Infectious Diseases Research Centre, Winnipeg, MB, Canada; ^3^Department of Microbiology and Veterinary Public Health, Chittagong Veterinary and Animal Sciences University, Chittagong, Bangladesh; ^4^National Microbiology Laboratory, Public Health Agency of Canada, Winnipeg, MB, Canada; ^5^Department of Biosystems Engineering, University of Manitoba, Winnipeg, MB, Canada; ^6^Department of Physics and Astronomy, University of Manitoba, Winnipeg, MB, Canada; ^7^Department of Biochemistry and Medical Genetics, University of Manitoba, Winnipeg, MB, Canada; ^8^Department of Immunology, University of Manitoba, Winnipeg, MB, Canada

**Keywords:** TILRR, pro-inflammatory cytokines/chemokines, cervical epithelial cell culture supernatants, THP-1, MOLT-4, Transwell assay, microfluidic device

## Abstract

TILRR has been identified as an important modulator of inflammatory responses. It is associated with NF-κB activation, and inflammation. Our previous study showed that TILRR significantly increased the expression of many innate immune responsive genes and increased the production of several pro-inflammatory cytokines/chemokines by cervical epithelial cells. In this study, we evaluated the effect of TILRR-induced pro-inflammatory cytokines/chemokines on the migration of immune cells. The effect of culture supernatants of TILRR-overexpressed cervical epithelial cells on the migration of THP-1 monocytes and MOLT-4 T-lymphocytes was evaluated using Transwell assay and a novel microfluidic device. We showed that the culture supernatants of TILRR-overexpressed HeLa cells attracted significantly more THP-1 cells (11–40%, *p* = 0.0004–0.0373) and MOLT-4 cells (14–17%, *p* = 0.0010–0.0225) than that of controls. The microfluidic device-recorded image analysis showed that significantly higher amount with longer mean cell migration distance of THP-1 (*p* < 0.0001–0.0180) and MOLT-4 (*p* < 0.0001–0.0025) cells was observed toward the supernatants of TILRR-overexpressed cervical epithelial cells compared to that of the controls. Thus, the cytokines/chemokines secreted by the TILRR-overexpressed cervical epithelial cells attracted immune cells, such as monocytes and T cells, and may potentially influence immune cell infiltration in tissues.

## Introduction

Previous studies identified that TILRR (Toll-like/Interleukin-1 receptor regulator), a FREM1 isoform 2, is an important regulator of genes in the NF-κB signal transduction pathway and inflammatory responses (Zhang et al., 2010, 2012). TILRR is expressed in human peripheral blood mononuclear cells, including monocytes and macrophages, and a wide range of human and mouse lymphocytic and mesenchymal cell lines (Zhang et al., 2010; Smith et al., 2017). It has been shown to cause aberrant inflammatory reactions and inflammation-driven pathological condition (Smith et al., 2017). Our previous study showed that the minor allele of FREM1 SNP (single nucleotide polymorphism) rs1552896 is associated with resistance to HIV-1 infection in the Pumwani sex worker cohort (PSWC) (Luo et al., 2012). Our group also showed that FREM1 is highly expressed in human epithelial tissues and immune cells, such as cervical tissues (Luo et al., 2012), CD4+ and CD8+ T cells, B cells, monocytes and natural killer (NK) cells (Omange et al., under revision). Recently, we have shown that TILRR modulates expression of many inflammation responsive genes and production of pro-inflammatory cytokines/chemokines such as IL-6, IL-8/CXCL8, IP-10/CXCL10, MCP-1/CCL2, MIP-1β/CCL4 and RANTES/CCL5 in cervico-vaginal epithelial cells (Kashem et al., 2019). The inflammatory cytokines/chemokines secreted by the TILRR-overexpressed cervical epithelial cells could influence immune cell migration to tissues. In fact, several studies have shown that chemo-attractants including IL-8/CXCL8, IP-10/CXCL10, MCP-1/CCL2, MIP-1α/CCL3, MIP-1β/CCL4, MIP-3α/CCL20, and RANTES/CCL5 produced by female genital epithelium induced rapid influx of circulating immune cells, resulting in increased risk of HIV acquisition (Mueller and Strange, 2004; Wira et al., 2005; Li et al., 2009; Masson et al., 2015; Arnold et al., 2016). Because cervical epithelial cells express FREM1 (Luo et al., 2012), and TILRR overexpression increases the production of pro-inflammatory mediators by cervical epithelial cell lines (Kashem et al., 2019), we hypothesized that the cytokines/chemokines produced by the cervical epithelial cell line may influence the migration and tissue infiltration of immune cells. In this study, we investigated the effect of supernatants of TILRR-overexpressed cervical epithelial cells (HeLa cells) on the migration of immune cells using two cell lines, THP-1 and MOLT-4, by Transwell assay and a novel microfluidic device that can record cell migration images. We report here that the supernatants of TILRR-overexpressed HeLa cells significantly attracted THP-1 (monocyte) and MOLT-4 (T-lymphocyte) cells than the controls.

## Materials and Methods

### Cell Lines and Culture Conditions

THP-1 (ATCC) (NIH, Catalog# 9942), a human monocytic cell line, and MOLT-4 (NIH, Catalog# 175), a human T lymphoblastic cell line, were used for *in vitro* migration assay. MOLT-4 cells were maintained in complete RPMI 1640 growth medium (Sigma-Aldrich, Catalog# R0883) supplemented with 10% fetal bovine serum (FBS) (Giboco, Catalog# 12483-020), 2 mM GlutaMax-I (Gibco, Catalog# 35050-061), 10 mM HEPES (Gibco, Catalog# 15630-080), 1 mM sodium pyruvate (Gibco, Catalog# 11360070), and 1% Pen-Strep (Gibco, Catalog# 15140-122). THP-1 cells were also maintained in complete RPMI 1640 growth medium similar to the MOLT-4 cells with additional supplement of 0.05 mM 2-Mercaptoethanol (Sigma-Aldrich, Catalog# M3148). The medium was replaced every 2–3 days. Because THP-1 (monocytes) and MOLT-4 (lymphocytes) cells express HIV-1 receptor/co-receptors CD4, CCR5 and CXCR4 essential for R5- and X4- tropic HIV-1 strains to infect the host (Dejucq et al., 1999; Dejucq, 2000; Konopka and Duzgunes, 2002; Miyake et al., 2003; Melo et al., 2014; Huang et al., 2016), and these cells are widely used as *in vitro* model for HIV-1 infection (Ushijima et al., 1991; Dejucq et al., 1999; Konopka and Duzgunes, 2002; Blanco et al., 2004; Cassol et al., 2006; Guo et al., 2014; Lodge et al., 2017), we therefore utilized these cell lines as a model for *in vitro* cell migration assay. HeLa cells (NIH, Catalog# 153) were maintained as described in our earlier study (Kashem et al., 2019). Briefly, the cells were cultivated in Dulbecco’s Modified Eagle’s Medium (DMEM) (Sigma-Aldrich, Catalog# D5796) supplemented with 10% FBS (Gibco, Catalog# 12483-020) and 1% Antibiotic-Antimycotic (Gibco, Catalog# 15240062). HeLa cells were used to produce cell culture supernatants following overexpression of TILRR. As human cervical tissues highly express FREM1 mRNA and TILRR is a transcript variant of FREM1, we therefore used HeLa cells as a model system to study the effect of FREM1 variant TILRR in promoting migration of immune cells.

### Overexpression of TILRR in HeLa Cells

We overexpressed the TILRR in HeLa cells as described previously (Kashem et al., 2019). In brief, approximately 2.5 × 10^5^ cells/ml was plated into each well of a 12-well culture plate containing complete DMEM growth medium a day before transfection. Once the cells reached 80–90% confluency, the media was replaced with antibiotic free fresh growth media. Overexpression of TILRR was performed by using 1.0 μg/well of TILRR-plasmid (vector + TILRR) (GeneCopoeia, Catalog# EX-I2135-68) or empty vector-plasmid control (GeneCopoeia, catalog# EX-NEG-68) containing a CMV promoter, an ampicillin marker, and a puromycin marker. We co-transfected the cells with 0.2 μg/well of PmaxGFP (Lonza, Walkersville, MD, United States) as a standard enhanced GFP (Green fluorescence protein) control vector to monitor the transfection efficiency by Confocal microscopy and Flow Cytometry analysis. Cells were co-transfected by 2 μl/well of EndofectinMax transfection reagent (GeneCopoeia, Catalog# EFM1004-01).

### Collection of Cervical Epithelial Cell Culture Supernatants

Secretion of inflammatory mediators from female genital epithelial cells demonstrated a critical role in rapid influx of immune cells at mucosal epithelia, resulting in heightened inflammation and vaginal microbial infection including HIV-1 (Fichorova et al., 2001; Kaul et al., 2008a, b; Li et al., 2009; Kaul et al., 2015). Thus, to mimic the physiological conditions of cervical epithelial microenvironment, TILRR-transfected HeLa cell culture supernatants were used as chemo-attractants in this study to investigate the effect on the migration of THP-1 monocytes and MOLT-4 lymphocytes. Culture supernatants from HeLa cells were produced as previously described (Kashem et al., 2019). Briefly, co-transfected HeLa cells were selected with puromycin treatment after 24 h of transfection. Cells were then incubated with FBS- and antibiotic-antimycotic free DMEM medium (Sigma Aldrich, Catalog# D5796) for another 24 h and the supernatants were collected in sterile centrifuge tubes. The culture supernatants were centrifuged at 10,000 × *g* for 10 min at 4°C, aliquoted in protein low binding tubes (Thermofisher Scientific, Catalog# 90410), and stored at −80°C for downstream experiments.

### Preparation of Cell Culture Supernatants

Immediately before the assay, cell culture supernatants were pulled from −80°C freezer and kept on ice to thaw. All samples were kept on ice until the assay plate was ready to use. The samples were vortexed for 15 s before being added to the plate. One freeze-thaw cycle was allowed for all culture supernatants to minimize sample degradation.

### Bio-Plex Analysis of Culture Supernatants

We analyzed the cytokines/chemokines in HeLa cell culture supernatants using a custom 13-plex panel as previously described (Kashem et al., 2019). These cytokines/chemokines include granulocyte macrophage colony stimulating factor (GM-CSF), interferon gamma (IFNγ), interleukin (IL)-1β, IL-6, IL-8/CXCL8, IL-10, IL-17A, IFN-γ inducible protein (IP)-10/CXCL10, macrophage chemo-attractant protein (MCP)-1/CCL-2, monocyte inflammatory protein (MIP)-1α, MIP-1β, regulated upon activation, normal T cell expressed and secreted (RANTES)/CCL5, and tumor necrosis factor alpha (TNF-α) (Supplementary Table 1). Briefly, the assay was conducted according to the Bio-Plex Pro^TM^ assays protocol (Bio-Rad Laboratories Inc.). Antibody-coupled bead stocks were vortexed for 15 sec and combined at 1:600 dilutions in assay buffer (Bio-Plex Pro^TM^ reagent kit, Bio-Rad, Catalog# 171-304070M). Fifty microliter of diluted beads was added into each well of 96-well Bio-Plex Pro^TM^ Flat bottom plate (Bio-Rad, Catalog# 171025001). After 2x washes with Bio-Plex wash buffer (BioRad, Catalog# 171-304070M), 50 μl of HeLa cell culture supernatants was added to the plate, and incubated for 30 min on plate shaker (850 ± 50 rpm) at room temperature (RT). Following incubation, the plate was washed 3X with wash buffer and 25 μl of detection antibody (1 μg/ml) was added into each well. The plate was incubated again for 30 min on a plate shaker. Fifty microliter of streptavidin-PE (1x) conjugate (BioRad, Catalog# 171304501) was added per well after 3X washes, and incubated for 10 min at RT. Finally, the plate was washed three times, and 150 μl of assay buffer was added into each well, shaken for 10 s and then run by Bio-Plex^TM^ 200 System (Luminex xMAP technology) (Bio-Rad, Canada). Complete HeLa cell growth medium was used as a diluents for Bio-Plex Pro Human Cytokine Standards Group I 27-plex (Bio-Rad, Catalog# 171D50001) and as a blank control. To generate standard curve, 50 μl of fourfold standard dilutions was added in 8-wells in duplicates. Bio-Plex software version 6.1 was used to acquire data, which was optimized to calculate the upper limit of quantification (ULOQ) (pg/ml) and lower limit of quantification (LLOQ) (pg/ml) using logistic-5PL regression analysis with fitness probability ≥ 0.95.

### Preparation of THP-1, and MOLT-4 -Cells for the Cell Migration Assays

The cell lines, following revival from the liquid nitrogen tank, were cultured for at least one passage before being used for the migration experiment. We used cells that were passaged for <10 times for this study. On the day of migration experiment, the cells were mixed gently and transferred to 50 ml BD Falcon tube, centrifuged for 10 min at 130 × *g* and the supernatants were discarded. Ten milliliters of RPMI 1640 complete medium without FBS and Pen-Strep was added to the pelleted cells, mixed gently, and the cell numbers were counted. For Transwell-based cell migration assay, a total of 5 × 10^5^cells/100μl/assay was used, whereas 1 × 10^4^ cells/10 μl/unit was used for microfluidic-based cell migration assay.

### Preparation of Positive Control Chemo-Attractants

Since HeLa cells were incubated with DMEM medium during the production of culture supernatants, DMEM medium was used as a medium control and diluent in this study. MCP-1/CCL2 (Sigma-Aldrich, Catalog# SRP3109-20UG) and stromal cell-derived factor (SDF)-1α/CXCL12 (Sigma-Aldrich, Catalog# SRP3276-10UG) were used as positive chemo-attractant controls for the migration assays. Positive chemo-attractant controls were diluted to concentrations (5, 10, 50, 100, and 200 ng/ml) with DMEM medium (Sigma-Aldrich, Catalog# D5796) without FBS and antibiotic-antimycotic for the optimization assay.

### Cell Migration Experiments in Transwell

Migration of cells was performed using 24-well polycarbonated membrane insert with 5 μm pore size (Corning, catalog# CLS3421) (Supplementary Figure 1). In the bottom chamber of Transwell plate, 600 μl of each chemo-attractant (DMEM control, diluted positive controls, culture supernatants of TILRR-overexpressed HeLa cells, culture supernatants of empty plasmid-transfected HeLa cells, or culture supernatants of non-transfected HeLa cells) was added. One hundred microliter of cells (5 × 10^5^ cells) in RPMI 1640 migration media was added to the upper chamber (Transwell insert), and incubated for 24 h at 37°C with 5% CO_2_. The number of input cells was calculated using three different counting methods, such as hemocytomer, automated cell counter (Invitrogen, Catalog# C10227), and flow cytometry (BD accuri C6, BD Biosciences, CA, United States). After 24 h of migration, Transwell insert was carefully removed from the well, and the medium containing the migrated cells in the bottom chamber was gently mixed and transferred to the 1.5 ml Eppendorf tube and migrated cells in the 50 μl medium were counted using hemocytometer and automated cell counter as described previously (Louis and Siegel, 2011; Cadena-Herrera et al., 2015; Pioli, 2019). For flow cytometry counting, the remaining ∼550 μl volume of the medium containing the migrated cells was gently vortexed and analyzed with BD accuri C6 (BD Biosciences, CA, United States). The data obtained from flow cytometry were analyzed with FlowJo software (Treestar, United States).

### Preparation of the Microfluidic Device

**FIGURE 1:**
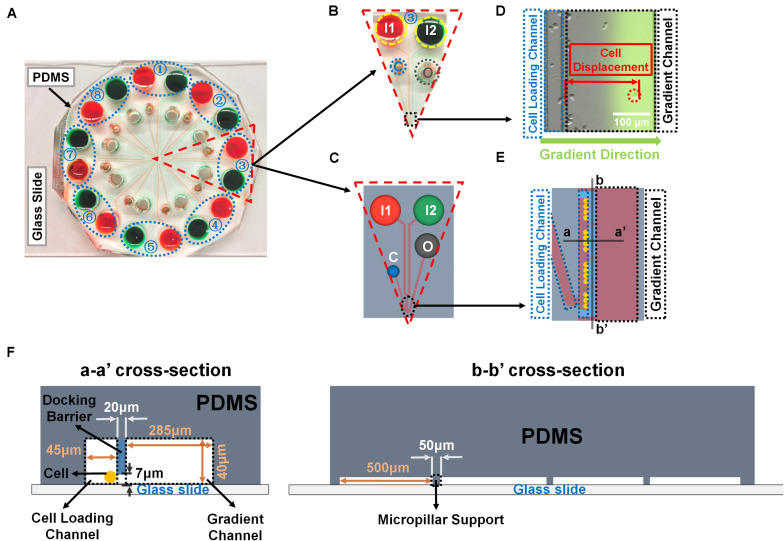
Illustration of the radial microfluidic device and cell migration analysis. **(A)** A representative image of the real radial microfluidic device with colored dyes to show the major networks in each unit. The upper transparent part is PDMS replica, which is bonded onto the bottom part of the glass slide; the blue elliptical-dashed box and the number number (1–8) show the chemical inlets of each independent unit. **(B,C)** Magnified view of the selected unit 3 of A in real device B and in schematic diagram C. I1 and I2: chemical inlets; C: cell loading port; O: waste outlet. **(D)** A representative experimental image to illustrate the data analysis of cell migration displacement in the magnified view of selected black-dashed box in B. **(E)** Magnified view of the black elliptical-dashed box of schematic C. In D and E, the blue- and black-dashed boxes represent the cell loading and gradient channels, respectively; the green arrow indicates the gradient direction; red arrow shows the displacement of an individual migrated cell. **(F)** Cross sectional views to illustrate the detailed designs of the device as indicated in E.

A previously designed radial microfluidic device was used in this study (Figure 1) (Wu et al., 2018). The device consisted of two layers with different thickness, the first layer (∼7 μm high) forms the cell docking structure to trap cells inside the cell loading channels; while the second layer (∼40 μm high) includes the cell loading ports and channels, and the gradient channels with chemical inlets and waste outlets. This device contains eight independent units, each one has its own two chemical inlets, one waste inlet, and one cell loading port, which allows eight independent experiments performed simultaneously. The device was fabricated by using previously described standard photolithography and soft lithography procedures (Wu et al., 2018; Ren et al., 2019, 2020). Briefly, the device pattern was designed by AUTOCAD and printed onto a transparent film at 24,000 dpi resolution (Fineline Imaging) served as the photomask for later photolithography. The pattern was then replicated by selected exposure of UV light through the photomask on top of a 3 inch silicon wafer (Silicon, Inc., ID, United States) with pre-coat of SU-8 negative photoresist (MicroChem). The wafer with patterns was used as the mold to reproduce polydimethylsiloxane (PDMS) (Sylgard 184, Dow Corning, Manufacturer SKU# 2065622) replicas, and then the replicas were cut off from the mold after 2 h of baking at 80°C. The chemical inlets (6 mm diameter), waste outlets (4 mm diameter), and cell loading ports (2 mm diameter) were punched out of the PDMS replica, and the replica was bonded onto a glass slide after air plasma treatment. The design of micropillar supports below the docking barrier increased the structural stability during bonding process. The device channels were coated with rat-tail collagen type I (20 μg/mL; Corning, Catalog# 354236) for 1 h, and then incubated with DMEM medium for another 30 min inside the incubator before the cell migration experiments.

### Cell Migration Experiments With the Microfluidic Device

Cells and chemo-attractants were prepared as described above. Cells were loaded into the cell loading ports. Fluorescein isothiocyanate (FITC)-dextran (10 kDa, Sigma-Aldrich, Catalog# FD10S) was added into the chemo-attractant solutions to indicate the gradient profile. DMEM medium and chemo-attractants were injected into the chemical inlet I1 and I2, respectively, to generate the gradient (Figures 1A–C). In addition, the two chemical inlets of each unit were covered by silicone oil (Alfa Aesar, Tewksbury, United States, Catalog# A12728-22) in order to balance the pressure difference for better gradient generation as previously described (Wu et al., 2018). The device was then placed under an inverted fluorescence microscope (Nikon Ti-U) inside an environmental controlled chamber (*InVivo* Scientific) at 37°C. Differential interference contrast (DIC) images of cell migration were taken for all the units at 0 and 24 h, respectively. The microfluidic device was incubated inside the incubator when not taking images. The DIC images of cell migration were obtained using NIS Element Viewer (Nikon) and ImageJ software (NIH). Specifically, cells that migrated away from the boundary of docking barrier to the gradient direction inside the gradient channel within the microscope field were recorded, and the displacement of each targeted cell was measured in each group at the end of the experiment using ImageJ (Figures 1D–F).

### Data Analysis

The data obtained with the Transwell assay was analyzed by GraphPad Prism software, version 8.3.0 (GraphPad Software, Inc., United States). The cell migration data obtained with the microfluidic device was processed by the OriginPro software. Each condition of experiment in this study was independently repeated at least three times. The statistical significant difference (*p*-value) between the treatment and control groups was determined by Student’s *t*-test.

## Results

### Optimization of Transwell Cell Migration Assay

**FIGURE 2:**
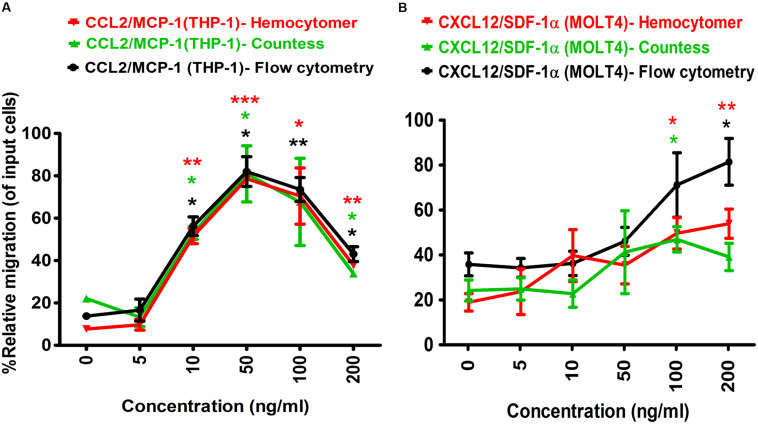
Optimization of positive control chemo-attractants for monocytes and lymphocytes migration using Transwell assay. **(A)** THP-1 monocytes, and **(B)** MOLT-4 T-lymphocytes migration was optimized using known chemo-attractants of MCP-1/CCL2 and SDF-1α/CXCL12, respectively. Chemo-attractants were diluted in DMEM without FBS and antibiotic-antimycotic. Approximately 5.0 × 10^5^ cells were used as input cells on upper chamber (insert) of Corning 24-well plate with 5.0 μm pore size polycarbonated membrane and incubated for 24 h. The line graph represents three independent experiments (mean ± SEM) compared to the DMEM base control (0 ng/ml chemo-attractants). Color-coded lines show the different counting methods. Red line and red asterisk’ represents hemocytometer count, green line with green asterisk’ for automated countess, and black line with black asterisk’ indicates flow cytometry count. Statistical comparisons conducted using Student’s *t*-test with 95% CI, all *p* < 0.05 were reported and indicated using an asterisks’ *p < 0.05, **p < 0.01, and ***p < 0.001. Legend on the top of the figures represents the chemo-attractants, type of cell used and counting method. *X*-axis shows the different concentrations of MCP-1/CCL2 and SDF-1α/CXCL12.

To determine the optimal concentration of positive control chemo-attractants for inducing chemotaxis of the monocytes and lymphocytes in Transwell assay, a series of dilution experiments were conducted using MCP-1/CCL2 and SDF-1α/CXCL12. One hundred microliter of 5 × 10^5^ cells diluted in RPMI 1640 medium was seeded into the apical chamber (insert) of Transwell plate, and allowed for migration at 37°C for 24 h. The results showed that THP-1 monocyte migration was gradually increased with increasing MCP-1/CCL2 concentration, and migration efficiency was gradually decreased to close to baseline level after 50 ng/ml MCP-1/CCL2 (Figure 2A). The highest percentage of THP-1 cell migration was observed at 50 ng/ml chemokine concentration, and the lowest percentage was with 5 ng/ml of MCP-1/CCL2 using three different cell counting methods. More specifically, the percentage of relative migration (PRM) was observed at 10 ng/ml (PRM: 51.53 ± 5.05%, *p* = 0.0077), 50 ng/ml (PRM: 78.57 ± 1.44%, *p* = 0.0007), 100 ng/ml (PRM: 70.41 ± 18.76%, *p* = 0.0424), and 200 ng/ml (PRM: 37.76 ± 1.45%, *p* = 0.0037) compared to the DMEM only control (PRM: 7.65 ± 2.16) by counting with hemocytometer. Similar migration behavior was also observed with automated cell counter, and flow cytometry analysis [10 ng/ml (PRM: 52.94 ± 4.16%, *p* = 0.0111; and PRM: 56.22 ± 6.18%, *p* = 0.0110), 50 ng/ml (PRM: 80.89 ± 18.72%, *p* = 0.0476; and PRM: 82.01 ± 9.92%, *p* = 0.0106), and 200 ng/ml (PRM: 33.82 ± 2.08%, *p* = 0.0299; and PRM: 43.02 ± 4.96%, *p* = 0.0153) of MCP-1/CCL2, respectively]. The highest PRM at 50 ng/ml of MCP-1/CCL2 was consistently determined with all three methods. MCP-1/CCL2 concentration higher than the 50 ng/ml diminished the effect of attracting THP-1 cells, and at 200 ng/ml the PRM was close to the baseline. Thus, 50 ng/ml of MCP-1/CCL2 is the best concentration to induce maximum percentage of THP-1 cells migration in conventional Transwell migration assay.

Similarly, the optimal concentration of SDF-1α/CXCL12 for MOLT-4 T-lymphocyte migration in Transwell assay was also determined (Figure 2B). The PRM of MOLT-4 T-lymphocyte significantly increased at 100 ng/ml (PRM: 49.64 ± 9.89%, *p* = 0.0184), and 200 ng/ml (PRM: 53.90 ± 9.19%, *p* = 0.0098) of SDF-1α/CXCL12 compared to that of DMEM control by counting with hemocytometer. MOLT-4 T-cell was also significantly migrated at 100 ng/ml (PRM: 46.92 ± 7.98%, *p* = 0.0359) and 200 ng/ml (PRM: 81.41 ± 14.70%, *p* = 0.0169) with automated cell counter and flow cytometry analysis, respectively. However, the PRM of MOLT-4 was reduced at 200 ng/ml of SDF-1α/CXCL12 by counting with automated cell counter. Because MOLT-4 T-cell significantly migrated at 100 ng/ml of SDF-1α/CXCL12, and the rate of migration was declined at 200 ng/ml by automated cell counter, we selected 100 ng/ml as the optimal concentration to induce maximum percentage of MOLT-4 cell migration in Transwell migration assay. This concentration was also found to be the best in inducing T-lymphocyte migration in earlier studies (Ottoson et al., 2001; Okabe et al., 2005; Tan et al., 2006). Taken together, the optimal concentration of both MCP-1/CCL2 and SDF-1α/CXCL12 were used as positive controls in Transwell and microfluidic device migration assays.

### TILRR Overexpression in HeLa Cells Significantly Induced Cytokines/Chemokines Production in Cell Culture Supernatants

To assess whether TILRR overexpression induces the production of cytokines/chemokines in cervical epithelial cells, we transfected HeLa cells with TILRR plasmid DNA as described elsewhere (Kashem et al., 2019). Bio-Plex analysis of HeLa cell culture supernatants demonstrated that TILRR significantly induced the production of IL-6, IL-8/CXCL8, IP-10/CXCL10, RANTES/CCL5 and MCP-1/CCL2 following 24h of incubation in the absence or presence of IL-1β stimulation compared to that of controls (Supplementary Figure 2). Thus, overexpression of TILLR in HeLa cells potentiates the production of soluble inflammatory mediators.

### The Culture Supernatants of TILRR-Transfected HeLa Cells Significantly Induced Migration of THP-1 Cells in Transwell Assay

**FIGURE 3:**
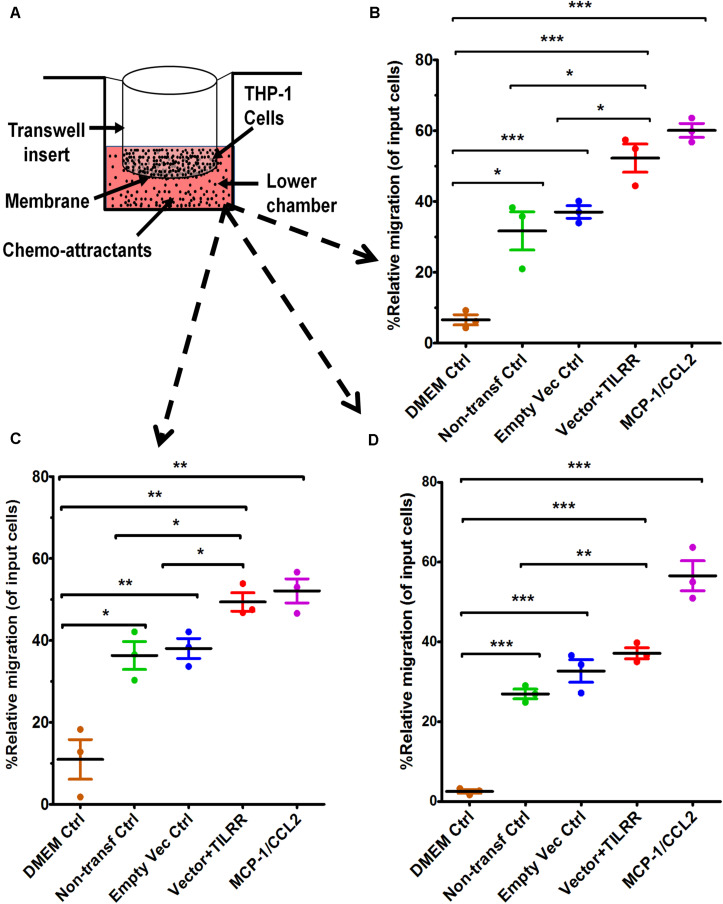
THP-1 monocytes migration toward TILRR-modulated cervical cell culture supernatants in Transwell assay. **(A)** Representative image of polycarbonated membrane Transwell unit. Approximately 5.0 × 10^5^ THP-1 cells were used as input cells on the upper chamber, and 600 μl of each chemo-attractant was dispensed in the bottom chamber of Transwell plate as described in materials and methods section. MCP-1/CCL2 (50 ng/ml) was used as a positive control chemo-attractant. Corning 24-well plate with 5.0 μm pore membrane used to conduct migration assay for 24 h. The migrated cells were counted with three independent counting methods, **(B)** hemocytometer, **(C)** automated countess, and **(D)** flow cytometry. Scatter dot plots represent the percent relative migration of cells in the presence of TILRR-transfected HeLa cell culture supernatants compared to the empty vector- and non-transfected control supernatants, and DMEM control. The data represent mean ± SEM of three independent experiments. Statistical comparisons conducted using Student’s *t*-test with 95% CI, all *p* < 0.05 were reported and indicated using an asterisks’ *p < 0.05, **p < 0.01, and ***p < 0.001. *X*-axis indicates the condition of chemo-attractants.

To examine if the presence of TILRR-modulated soluble cytokines/chemokines influences the migration of monocytes, Transwell migration assay was conducted using THP-1 monocytes and HeLa cell culture supernatants. The results demonstrated that TILRR-transfected HeLa cell culture supernatants attracted significantly higher amount of THP-1 cells (11–40%) than the controls (Figures 3A–D). Data analysis showed that approximately 16-46% higher amount of THP-1 cells were migrated toward the culture supernatants of TILRR-overexpressed HeLa cells compared to the supernatants of empty vector-transfected (PRM: 52.26 ± 6.88% vs. 37.04 ± 3.09%, *p* = 0.0250), non-transfected (PRM: 52.26 ± 6.88% vs. 31.69 ± 9.35%, *p* = 0.0373) HeLa cell supernatants, or DMEM controls (PRM: 52.26 ± 6.88% vs. 6.58 ± 2.49%, *p* = 0.0004) (hemocytometer counts) (Figure 3B). Similarly, the migration of significantly higher amount of THP-1 cells (12–39%) was also observed toward TILRR-transfected culture medium than the empty vector-transfected (PRM: 49.39 ± 3.90% vs. 38.04 ± 4.22%, *p* = 0.0267), non-transfected (49.39 ± 3.90% vs. 36.32 ± 5.90%, *p* = 0.0328) HeLa cell supernatants, or DMEM controls (49.39 ± 3.90% vs. 10.96 ± 8.39%, *p* = 0.0020) (automated cell counter methods) (Figure 3C). Flow cytometry analysis also showed that the higher numbers of THP-1 cells migrated toward TILRR-transfected culture medium (11-35%) than the controls (Figure 3D). Thus, the supernatants of TILRR-overexpressed HeLa cells attracted the THP-1 monocytes.

### The Culture Supernatants of TILRR-Transfected HeLa Cells Significantly Induced Migration of MOLT-4 T-lymphocytes in Transwell Assay

**FIGURE 4:**
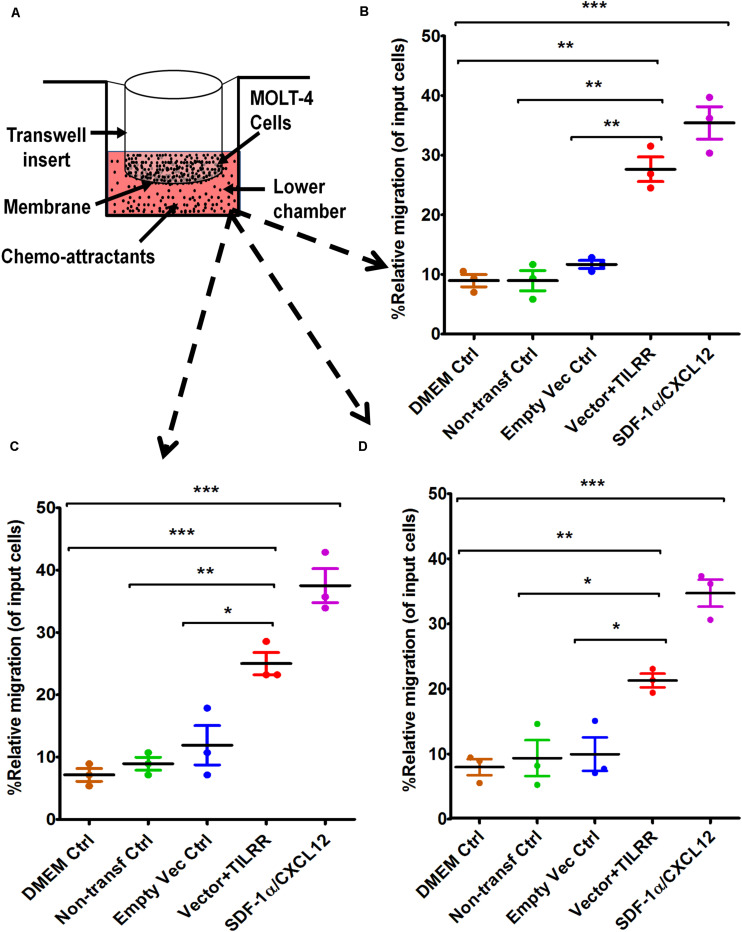
MOLT-4 T-lymphocytes migration toward TILRR-modulated cell culture supernatants in Transwell assay. **(A)** Representative image of polycarbonated-membrane Transwell unit. Approximately 5.0 × 10^5^ MOLT-4 lymphocytes cells were used, and 600 μl of culture media or positive control chemo-attractant slowly dispensed into the Transwell plate (bottom chamber) as described in materials and methods section. SDF-1α/CXCL12 (100 ng/ml) was used as a positive control. Similar Transwell plate (as THP-1) used to conduct migration of MOLT-4 cells for 24 h. The migrated cells were counted with three independent counting methods such as **(B)** hemocytometer, **(C)** automated countess, and **(D)** flow cytometry. Scatter dot plots represent the percent relative migration of cells in the presence of TILRR-transfected HeLa cell culture supernatants compared to the empty vector- and non-transfected control supernatants, and DMEM control. The data represent mean ± SEM of three independent experiments. Statistical comparisons conducted using Student’s *t*-test with 95% CI, all *p* < 0.05 were reported and indicated using an asterisks’ *p < 0.05, **p < 0.01, and ***p < 0.001. *X*-axis indicates the condition of chemo-attractants.

To assess whether the supernatants of TILRR-overexpressed HeLa cells attract T-lymphocytes, MOLT-4 cell migration was conducted using similar approach. MOLT-4 cells were also showed higher PRM (14-17%) toward TILRR-modulated HeLa cell culture supernatants compared to that of controls (Figures 4A–D). Approximately 16–19% higher amount of MOLT-4 cells were significantly attracted to TILRR-overexpressed HeLa cell supernatants than the supernatants of empty vector-transfected (PRM: 27.63 ± 3.57% vs. 11.67 ± 1.17%, *p* = 0.0018), non-transfected (PRM: 27.63 ± 3.57% vs. 8.95 ± 2.94%, *p* = 0.0022) cells, or DMEM control (PRM: 27.63 ± 3.57% vs. 8.95 ± 1.78%, *p* = 0.0013) (hemocytometer counting method) (Figure 4B). Similar trend of MOLT-4 cell migration was observed by automated cell counter and flow cytometry analysis where the PRM was observed approximately 14–18% and 12–14% higher in TILRR-overexpressed HeLa cell culture supernatants than that of controls, respectively (Figures 4C,D). Collectively, these data showed that the supernatants of TILRR-overexpressed HeLa cells also attract T-lymphocytes.

### The Culture Supernatants of TILRR-Transfected HeLa Cells Significantly Induced Migration of THP-1 Monocytes by the Microfluidic Device Analysis

**FIGURE 5:**
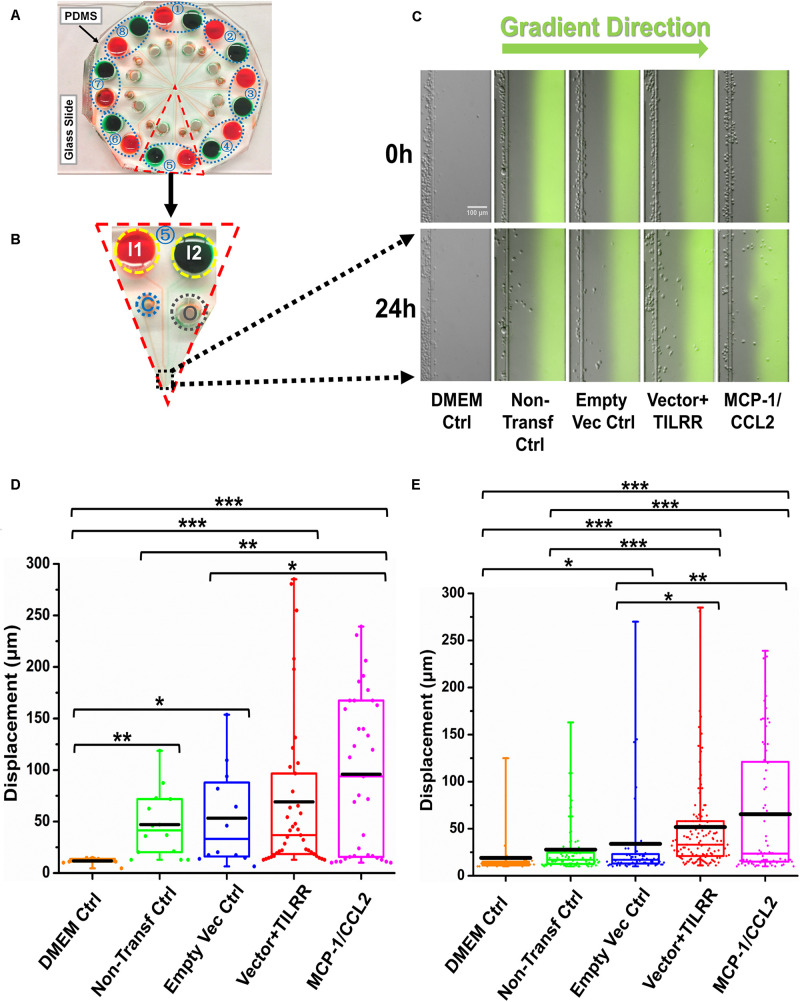
THP-1 monocytes migration toward TILRR-modulated cell culture supernatants in microfluidic device. **(A)** A representative image of the real radial microfluidic device with colored dyes to show the major networks in each unit. The upper transparent part is PDMS replica, which is bonded onto the bottom part of the glass slide; the blue elliptical-dashed box and the number (1–8) show the chemical inlets of each independent unit. **(B)** One magnified triangular unit (randomly selected) from A showing the black-dashed square area from where migrated cells were analyzed. I1 and I2: chemical inlets; C: cell loading port; O: waste outlet. **(C)** Representative images of the migration of THP-1 cells in different conditions at 0 and 24 h analyzed from B. Green color in the image of all experimental conditions except DMEM control indicates the profile and most concentrated area of chemical gradient. **(D)** The colored box plots show the total displacement of each cell in the corresponding experimental groups in C. **(E)** The colored box plots show the total displacement of each cell in three independent experiments. The top and bottom of the whisker show the maximum and minimum value; the box shows the migrated cells within the range from 25% to 75% of total cells based on the ranked displacement value; the black bold line indicates the mean displacement. The data in different groups were compared using Student’s *t*-test with 95% CI, all *p* < 0.05 were reported and indicated using asterisks’ *p < 0.05, **p < 0.01, and ***p < 0.001.

Because Transwell assay does not provide the data on how far (directional displacement) a cell can migrate toward the HeLa cell culture supernatants, we next sought to examine the displacement of THP-1 monocytes from their original location toward the gradient of culture supernatants by a novel microfluidic device. The microfluidic device offers better controlled chemical gradient with single cell analysis, which enables the data analysis of the displacement and distribution of individual migrated cell during the experiment. The results showed that TILRR-overexpressed HeLa cell culture supernatants attracted more THP-1 cells with significantly higher average cell displacement compared to the controls (Figures 5A–E). Furthermore, our data showed that THP-1 cells migrated further to the supernatants of TILRR-overexpressed HeLa cells in comparison to the supernatants of the empty vector-transfected HeLa cells (52.00 ± 52.00 vs. 34.00 ± 48.00 μm, *p* = 0.0180), non-transfected HeLa cells (52.00 ± 52.00 vs. 28.00 ± 29.00 μm, *p* < 0.0001), or DMEM control (52.00 ± 52.00 vs. 19.00 ± 23.00 μm, *p* < 0.0001) (Figure 5E). Thus, the TILRR-transfected HeLa cell supernatants promoted migration of THP-1 monocytes in microfluidic device assay.

### The Culture Supernatants of TILRR-Transfected HeLa Cells Significantly Induced Migration of MOLT-4 T-lymphocytes by the Microfluidic Device Analysis

**FIGURE 6:**
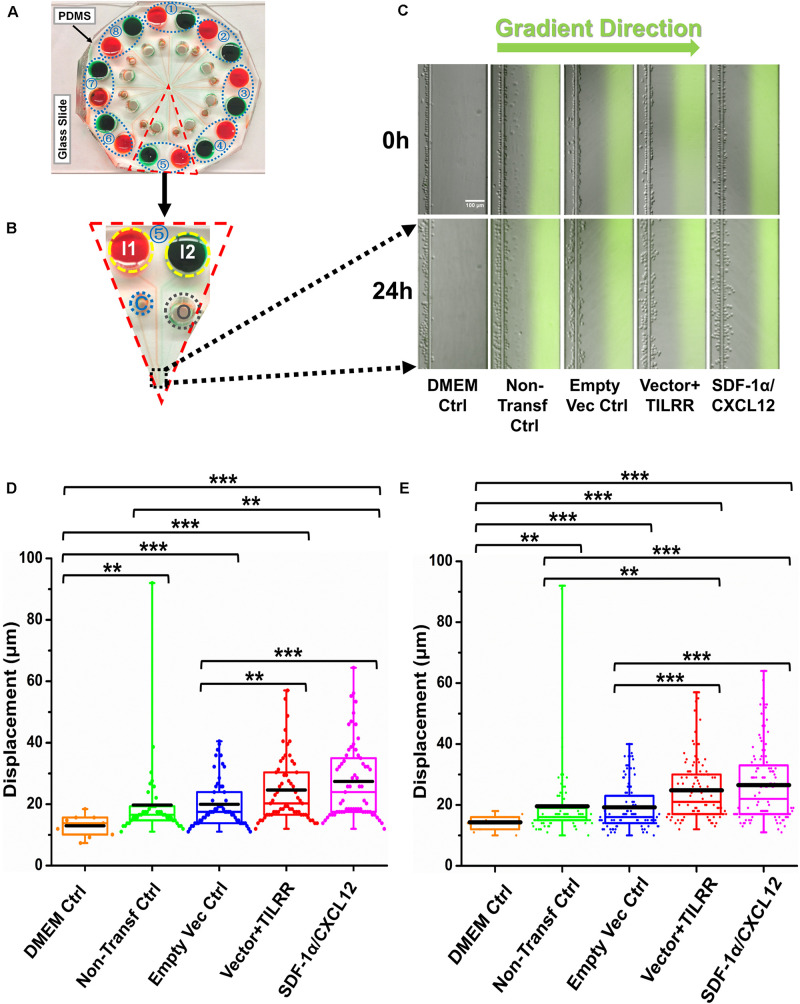
MOLT-4 T cells migration toward TILRR-modulated cell culture supernatants in microfluidic device. **(A)** A representative image of the real radial microfluidic device with colored dyes to show the major networks in each unit. The upper transparent part is PDMS replica, which is bonded onto the bottom part of the glass slide; the blue elliptical-dashed box and the number (1–8) show the chemical inlets of each independent unit. **(B)** One magnified triangular unit (randomly selected) from A showing the black-dashed square area from where migrated cells were analyzed. I1 and I2: chemical inlets; C: cell loading port; O: waste outlet. **(C)** Representative images of the migration of THP-1 cells in different groups at 0 and 24 h analyzed from B. Green color image of all experimental conditions except DMEM control indicates the profile and most concentrated area of chemical gradient. **(D)** The colored box plots show the total displacement of each cell in the corresponding experiment conditions in C. **(E)** The colored box plots show the total displacement of each cell in three independent experiments. The top and bottom of the whisker show the maximum and minimum value; the box shows the migrated cells within the range from 25% to 75% of total cells based on the ranked displacement value; the black bold line indicates the mean displacement. The data in different groups were compared using Student’s *t*-test with 95% CI, all *p* < 0.05 were reported and indicated using asterisks’ *p < 0.05, **p < 0.01, and ***p < 0.001.

We also evaluated the effect of the culture supernatants of TILRR-transfected HeLa cells on the migration of MOLT-4 T-lymphocyte with the microfluidic device. The results showed that TILRR-overexpressed culture supernatants also attracted more MOLT-4 cells with significantly higher average cell displacement (Figures 6A–E). Similar to the THP-1 cells, MOLT-4 T-lymphocytes migrated further toward the supernatants of TILRR-overexpressed HeLa cells compared to the supernatants of empty vector-transfected HeLa cells (25.00 ± 10.00 vs. 19.00 ± 8.00 μm, *p* < 0.0001), non-transfected (25.00 ± 10.00 vs. 20.00 ± 13.00 μm, *p* = 0.0025) HeLa cells, or DMEM control (25.00 ± 10.00 vs. 14.00 ± 3.00 μm, *p* < 0.0001) (Figure 6E). Thus, TILRR-transfected HeLa cell supernatants also promoted MOLT-4 T-lymphocytes migration with microfluidic device assay.

## Discussion

Our previous study showed that TILRR regulates the expression of many inflammation-responsive genes in NF-κB signaling pathways, and influences the production of multiple pro-inflammatory mediators in cervical and vaginal mucosal epithelial cell lines (Kashem et al., 2019). Because TILRR expression promoted the production of several cytokines/chemokines in cervical epithelial cell lines, we investigated the effect of the supernatants of the TILRR-overexpressed HeLa cells on the migration of immune cells in this study using Transwell and microfluidic device assays. Our data showed that the supernatants of TILRR-overexpressed HeLa cells attracted migration of THP-1 and MOLT-4 cells in Transwell assay. In addition, with a novel microfluidic assay, we demonstrated that the THP-1 and MOLT-4 cells migrated further toward the supernatants of the TILRR-overexpressed HeLa cells. THP-1 cells (monocytes) and MOLT-4 cells (T cells) are CD4-expressing cells, the targets of HIV-1 (Berger et al., 1998; Verani et al., 1998; Borrajo et al., 2019). Thus, the supernatants of TILRR-overexpressed HeLa cells, containing multiple pro-inflammatory cytokines (Kashem et al., 2019), attracts HIV-1 target cells.

The purpose of using two different cell migration techniques in this study was to confirm the study findings in parallel. Traditional Transwell assay was used to analyze the ability of the cells to migrate through a porous membrane accompanied by the relative percentage of total cell migration toward the gradients of HeLa cell culture supernatants. Traditional Transwell assay is convenient, and compatible with all kinds of cell types, and most widely used sensitive quantification of *in vitro* cell migration technique (Boyden, 1962). However, Transwell assay does not provide how far a single cell can migrate toward the added chemo-attractants. Despite the popularity and advantages of Transwell for cell migration study, this assay only offers the endpoint readout of entire number of cells without providing other important migratory aspects on individual cell level. In order to fill the gap and exclude the possible effect on the gravity force of loaded cells during experiment, we applied a novel microfluidic device to further confirm the attraction effect of the same supernatants on the migration of THP-1 and MOLT-4 cells. Specifically, this microfluidic device enables eight independent experiment groups conducted simultaneously, which dramatically increased the experimental throughput. Unlike other microfluidic device that requires the external input such as pumps to generate and maintain a stable chemical gradient, this device offers stable chemical gradient generated by the pressure differences between the chemical inlets and waste outlets in a well-controlled manner (Wu et al., 2018). In addition, the advanced cell docking design provides the initial alignment of all the loaded cells in the cell-loading channel at the beginning, which increases the accuracy of displacement measurement on each individual migrated cell during the experiment. On the other hand, the presence of the docking barrier requires all loaded cells to transform the cell size to squeeze and horizontally migrate through the area when stimulated by the chemical gradient, which excludes the gravity issue that may exist in Transwell assay. Microfluidic devices have been widely applied for cell migration studies because of several advantages, including extremely low cell and reagent consumption, stable chemical gradient generation, and live-cell tracking. Due to the versatile features of the microfluidic device, many important properties of individual migrated cell such as speed and displacement can be extracted from the migration experiment (Ren et al., 2017). Therefore, the combination of these two techniques in our experimental settings confirmed that immune cells, the HIV-1 target cells, were successfully migrated to the TILRR-overexpressed HeLa cell culture supernatants.

Studies have shown that pro-inflammatory cytokines/chemokines produced from female genital epithelial cells induce rapid influx of HIV-1 target cells leading to inflammation (Kaul et al., 2008a, b, 2015; Li et al., 2009; Haase, 2010). The elevated levels of cytokines/chemokines IL-8/CXCL8, MIP-1α/CCL3, and MIP-1β/CCL4 are strongly associated with the recruitment of monocytes/macrophages and neutrophils in female genital secretion (Fichorova et al., 2001). Our study showed that TILRR plays an important role in regulating this pro-inflammatory cytokine environment and immune cell infiltration in cervical epithelial tissue. TILRR could promote the migration of immune cells, especially the HIV-1 target cells, in the female genital epithelium through the modulation of pro-inflammatory cytokines/chemokines secretion, resulting in increased risk of vaginal HIV-1 infection and transmission. Thus, TILRR-promoted migration of immune cells warrants further studies.

While our *in vitro* study was the first to suggest the important role of TILRR in immune cell migration, the limitation of the current work is that it is the first insight based on *in vitro* systems and a follow up *in vivo* study will be an important future direction. Future studies using *in vivo* model, such as mouse model or macaque model (Rhesus macaque [*Macaca mulatta*]) will help to understand the novel role of TILRR in promoting migration of immune cells into genital epithelial tissues. The *in vivo* model(s) can further be combined with using human cervical biopsy samples. Collectively, these potential future studies as follow-up of the current work may identify new intervention technology to control TILRR-induced immune cell infiltration to the epithelial tissues, and inflammation-induced vaginal HIV-1 infection.

## Conclusion

TILRR-modulated cytokines/chemokines from HeLa cells promote the migration of immune cells. We are the first to show that TILRR can regulate migration of HIV-1 target cells into cervical epithelial tissues. Targeting TILRR may lead to new interventions in reducing vaginal HIV-1 infection.

## Data Availability Statement

All datasets generated for this study are included in the article/Supplementary Material.

## Author Contributions

FP and ML acquired the funding. MK, ML, and FL conceived and designed the research. MK and XR performed the research, analyzed the data, and wrote the manuscript. ML and FL supervised the research. ML, FL, HL, BL, and LL edited the manuscript. MK revised, formatted and submitted to the journal. All the authors have approved this manuscript.

## Conflict of Interest

The authors declare that the research was conducted in the absence of any commercial or financial relationships that could be construed as a potential conflict of interest.
