# Supplementary material for: TILRR Promotes Migration of Immune Cells Through Induction of Soluble Inflammatory Mediators
Source: Front Cell Dev Biol. 2020 Jul 3;8:563. doi: 10.3389/fcell.2020.00563 (PMC7348050; doi:10.3389/fcell.2020.00563)
Supplement: Supplementary file 1 [file Data_Sheet_1.PDF]

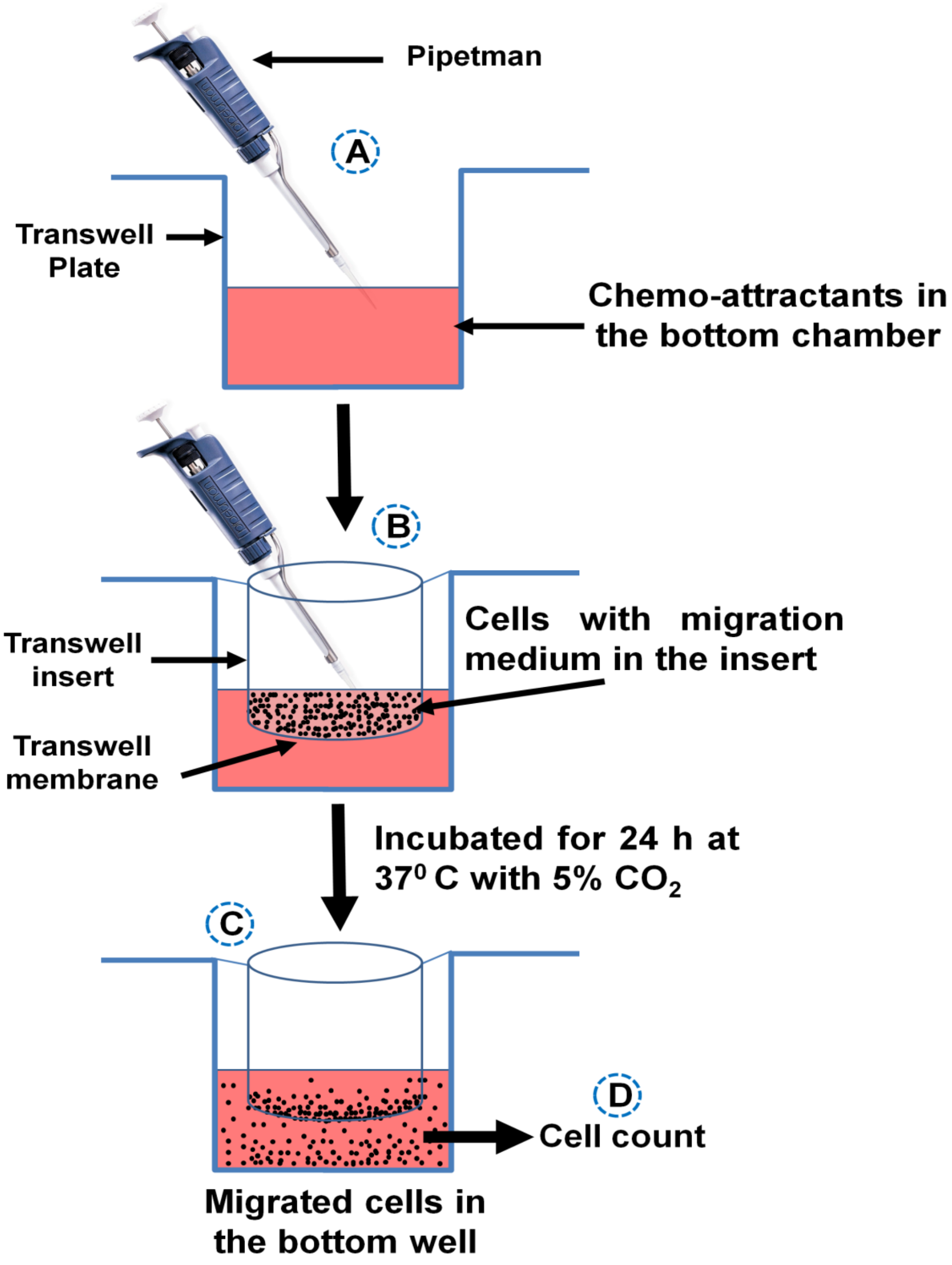

Supplementary Figure 1: Illustration of the Transwell migration assay. (A) TILRR-overexpressed HeLa cell culture supernatants (light red color) added to the bottom chamber of Tranwell plate. **(B)** Immune cells (5x10^5/insert) (THP-1 or MOLT-4) containing migration media dispensed in Transwell insert. **(C)** Following 24h of incubation at 37^0 C, the migrated cells in bottom chamber were examined under microscope. **(D)** Media containing migrated cells were collected from bottom chamber and estimated for cell number using hemocytometer, automated cell counter and flow cytometry analysis.

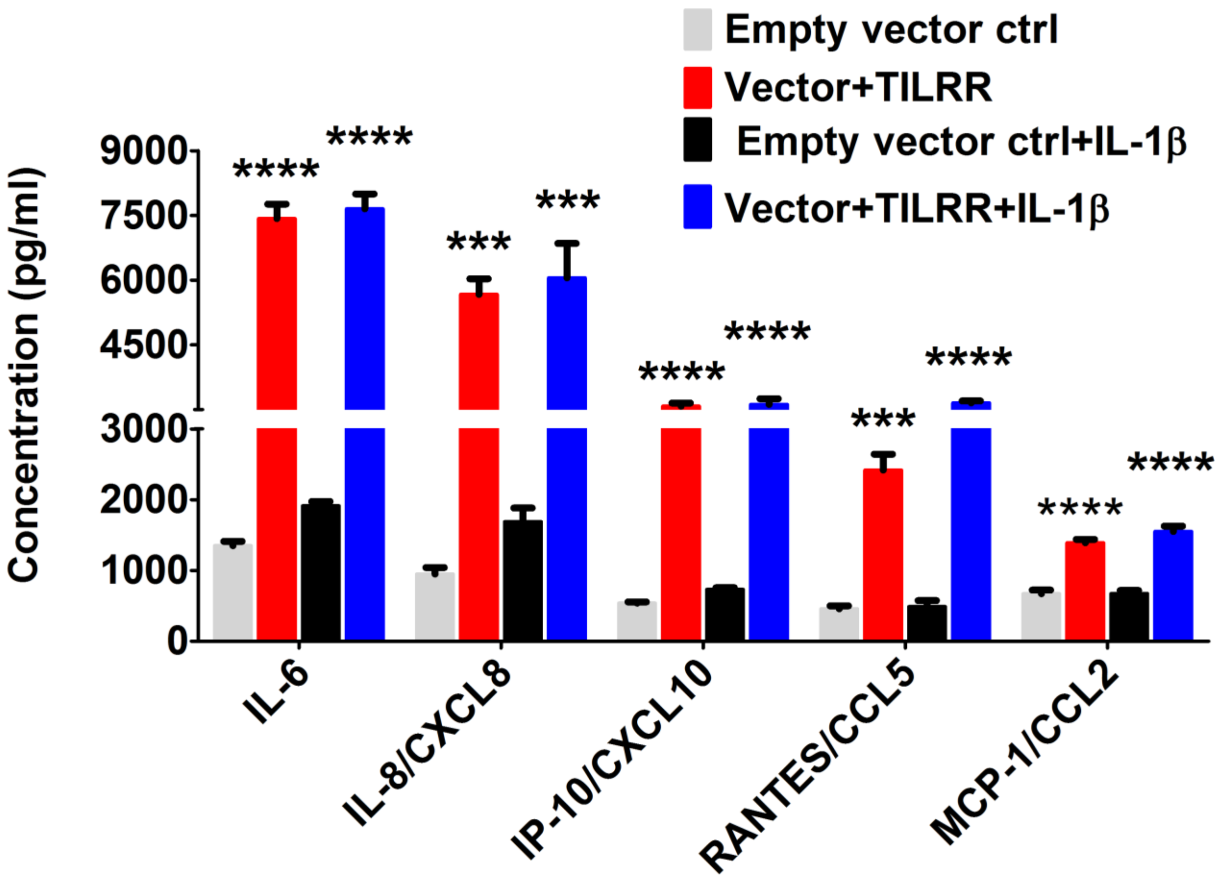

Supplementary Figure 2: TILRR overexpression in HeLa cells increased the production of Pro-inflammatory cytokine/chemokine(s) with or without added IL-1β. HeLa ($5x10^5$cells/well) cells were co-transfected with either pEZ-TILRR-M68 (1.0µg/$5x10^5$ cells) or pEZ-NEG-M68 (1.0µg/$5x10^5$ cells) with PmaxGFP (0.2µg/$5x10^5$ cells) vector as explained in materials and methods section. In parallel, cells were incubated with or without the addition of IL-1β (1nM) in serum free DMEM (HeLa) media and then the cultured media were collected. Thirteen different inflammatory cytokines were measured using an in-house developed multiplex cytokine/chemokine(s) bead assay with BioPlex 200 (BIORAD). The data represent the relative level of vector+TILRR, in the presence or absence of IL-1β, compared to the empty vector control. The sample measurements below the detection limit were assigned as zero. The data represent mean of three independent experiments (mean±SEM). Statistical comparisons conducted using student t test with 95% CI, all $p<0.05$ were reported and indicated using an asterisks' ***$p<0.001$, and ****$p<0.0001$. Legends on the upper right corner show the experimental conditions. The data were adapted from Kashem et al. (5) under the license CC BY 4.0.

Supplementary Table 1: Primary and secondary antibodies used in Bio-Plex Multiplex cytokines/chemokines bead assay

SL	Name	Catalog#	Vendor
**Primary antibody (Uncoupled)**			
**SL**	**Name**	**Catalog#**	**Vendor**
1	Human IL-1beta/ IL-1F2 Antibody	MAB601-500	R&D System
2	Human IL-6 MAb	M620	ThermoFisher Scientific
3	Rat Anti-Human IL-10-UNLB	10100-01	SouthernBiotech
4	Human/Primate IL-17/IL-17A Antibody	MAB317-500	R&D System
5	Human IP-10/ CXCL10/CRG-2 Antibody	MAB266-500	R&D System
6	Human CXCL8/IL-8 MAb	M801	ThermoFisher Scientific
7	Human CCL5/RANTES PAb	P230E	ThermoFisher Scientific
8	Rat Anti-Human GM-CSF-UNLB	10111-01	SouthernBiotech
9	Human IFNγ MAb	M700A	ThermoFisher Scientific
10	Human MCP-1/CCL2/JE Antibody	MAB679-500	R&D System
11	Human MIP-1α/CCL3Antibody	AF-270-NA	R&D System
12	Human MIP-1β/CCL4 Antibody	MAB271-100	R&D System
13	Human TNFα MAb	M303	ThermoFisher Scientific
**Detection antibody (Biotinylated)**			
**SL**	**Name**	**Catalog#**	**Vendor**
1	Human IL-1beta/IL-1F2 Biotinylated Antibody	BAF201	R&D System
2	Human IL-6 MAb, Biotin-labeled	M621B	ThermoFisher Scientific
3	Rat Anti-Human IL-10-BIOT	10110-08	SouthernBiotech
4	Human/Primate IL-17/IL-17A Biotinylated Antibody	BAF317	R&D System
5	Human IP-10/CXCL10/CRG-2 Biotinylated Antibody	BAF266	R&D System
6	Human CXCL8/IL-8 MAb, Biotin-labeled	M802B	ThermoFisher Scientific
7	Human CCL5/RANTES MAb, Biotin-labeled	M230B	ThermoFisher Scientific
8	Rat Anti-Human GM-CSF-BIOT	10112-08	SouthernBiotech
9	Human IFNγ MAb, Biotin-labeled	M701B	ThermoFisher Scientific
10	Human MCP-1/CCL2/JE Biotinylated Antibody	BAF279	R&D System
11	Human MIP-1α/CCL3 Biotinylated Antibody	BAF270	R&D System
12	Human MIP-1β/CCL4 Biotinylated Antibody	BAF271	R&D System
13	Human TNFα MAb, Biotin-labeled	M302B	ThermoFisher Scientific

This table is adapted from Kashem et al. (1) under the license CC BY 4.0

Reference

1. Kashem MA, Li H, Toledo NP, Omange RW, Liang B, Liu LR, et al. Toll-like Interleukin 1 Receptor Regulator Is an Important Modulator of Inflammation Responsive Genes. Frontiers in Immunology. 2019;10(272):1-16. DOI: 10.3389/fimmu.2019.00272.
